# Drowning - a scientometric analysis and data acquisition of a constant global problem employing density equalizing mapping and scientometric benchmarking procedures

**DOI:** 10.1186/1476-072X-10-55

**Published:** 2011-10-14

**Authors:** David A Groneberg, Ute Schilling, Cristian Scutaru, Stefanie Uibel, Simona Zitnik, Daniel Mueller, Doris Klingelhoefer, Beatrix Kloft

**Affiliations:** 1 Institute of Occupational, Social and Environmental Medicine, Goethe-University, Frankfurt, Germany; 2 Otto-Heubner-Centre, Charité-Universitätsmedizin Berlin, Berlin, Germany

**Keywords:** drowning, near-drowning, occupational medicine, scientometrics, density equalizing mapping

## Abstract

**Background:**

Drowning is a constant global problem which claims approximately half a million victims worldwide each year, whereas the number of near-drowning victims is considerably higher. Public health strategies to reduce the burden of death are still limited. While research activities in the subject drowning grow constantly, yet there is no scientometric evaluation of the existing literature at the present time.

**Methods:**

The current study uses classical bibliometric tools and visualizing techniques such as density equalizing mapping to analyse and evaluate the scientific research in the field of drowning. The interpretation of the achieved results is also implemented in the context of the data collection of the WHO.

**Results:**

All studies related to drowning and listed in the ISI-Web of Science database since 1900 were identified using the search term "drowning". Implementing bibliometric methods, a constant increase in quantitative markers such as number of publications per state, publication language or collaborations as well as qualitative markers such as citations were observed for research in the field of drowning. The combination with density equalizing mapping exposed different global patterns for research productivity and the total number of drowning deaths and drowning rates respectively. Chart techniques were used to illustrate bi- and multilateral research cooperation.

**Conclusions:**

The present study provides the first scientometric approach that visualizes research activity on the subject of drowning. It can be assumed that the scientific approach to this topic will achieve even greater dimensions because of its continuing actuality.

## Introduction

Down to the present day, drowning is still a constant global and underestimated problem with a variety of implications for public health [1]: There are around half a million victims worldwide each year, mostly in developing and industrializing countries [2]. Tragically, the problem of drowning was recently spotlighted in the public focus again because of the tragic seaquake in South East Asia in 2004 and the Subsaharian refugees drowning in the Mediterranean Sea.

Regarding the age pattern of drowning victims, there is a considerable accumulation among children [3]. Most people drown in their domestic surroundings like bath tubs and swimming pools, but also in the open sea [4]. The notably high prevalence of male drowning victims is based upon on a more risky behaviour and alcohol consumption while practicing water sports. Medical risk factors include epilepsy [5], coronary heart disease, cardioarrhythmias, myocardial infarction, ventricular fibrillation, cerebro-vascular damage [6] and long QT syndrome [7]. In contrast to prior scientific research results, hypoxia is nowadays known to be the most important pathophysiologic principle in the drowning process [8]. The complex of clinical symptoms is heterogeneous and varies individually: Pulmonary symptoms like acidosis, ARDS and aspiration pneumonia rank first, followed by neurological damage. Cardiovascular symptoms, changes in electrolyte and blood gases are not as dominant in the clinical picture as previously assumed [9]. The key to fewer drowning victims is public education about risks and development of multidimensional preventive measures [10]. Still drowning remains a constant problem of the global burden of disease [1]. Precise scientometric approaches have not been implemented so far to analyse worldwide research activities in the specific field of drowning. Hence, the present study analysed scientometric parameters in the field of drowning employing classical bibliometric techniques in combination with novel visualizing calculations and large databases. We aimed to assess certain parameters as stated below.

## Methods

### Data source and time span

The present approach bases on the NewQIS platform, which combines bibliometric with density-equalizing techniques in the field of biomedicine [11-14]. In brief, data was retrieved from one of the biggest scientific online database, the *Web of Science *from the *Thompson Scientific*, formerly known as *Institute of Scientific Information (ISI)*. The period 1900 to 2006 was used as restriction for the publication date since the data entry for 2007 had not been completed yet by the time of analysis. Exceptions were made wherever necessary and possible.

### General and specific search strategies

All published items including the search term "drowning" were selected and downloaded for analyzing purposes using a web interface. The total number of published items was 2381 (last update: 18.12.2007). Subsequent analysis on published items per year, country and institution, publication languages, journals and impact factor and authors followed.

Furthermore, the latest data about the subject of drowning retrieved from the World Health Organisation (WHO) were consulted for specific analysis. The most actual data available about total number of drowning deaths and worldwide drowning rates (drowning deaths per 100,000 inhabitants) originate from the year 2002 [15].

### Density equalizing mapping

Density equalizing mapping procedures were employed as described in previous studies [16,17]. All territories were correlated with different parameters and subsequently re-sized according to 1) the number of published items related to drowning, 2) worldwide drowning rates and 3) the total number of drowning deaths worldwide. The developing image of the world map, the cartogram, is distorted according to the corresponding parameter. The calculations of this procedure are based of Gastner's and Newman's algorithm [18].

### Analysis of country and author cooperation

An analysis of bilateral country cooperation was carried out to assess research networks. A bilateral cooperation between to countries was defined when at least one author originates from one country and at least one other author from a second country. A matrix with all identified countries was worked out and filled with the corresponding values for the cooperation for each pair of countries. A second software module was developed to translate the matrix and to transform the figures into vectors. The thickness and colour of a vector indicates the number of cooperation articles between the two countries. For a clear synoptic view, a threshold of at least ten cooperations was set for the multilateral country analysis. The results were visualized graphically employing the chart technique which was also used to display the author cooperation.

### Qualitative parameter analysis

Citation analyses provide a qualitative assessment of the quantitative data obtained. It is used as an indicator for research quality, followed by a careful approach to an interpretation with regards to contents and context. For all published items on drowning, the total number of citations and the citation rate (average citation per published article) was calculated. Also, a citation analysis per publication language was performed.

## Results

### Total number of published items and citations and citation rate

**Figure 1:**
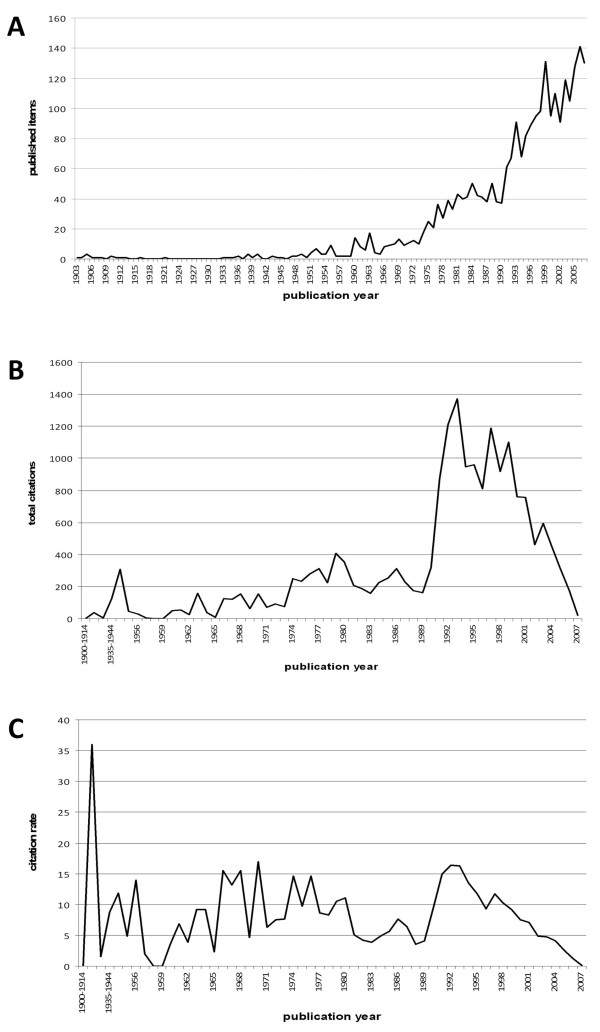
**Publication analysis.** (A) Analysis of total number of published items, (B) total number of citations, (C) citation rate per publication year.

The total number of published items on the subject of drowning was employed as an indicator of quantity of research productivity. In total, a number of 2381 articles were identified using the ISI-Web of Science database. Obviously, the publication effort has increased constantly in the past few years. The number of published items is considered to be an index of quantity of research productivity. The oldest publication on drowning listed is from the year 1903. A temporary maximum of 141 relevant articles is documented for the year 2006, a number of 130 for the year 2007. Around one third of all 2526 publications on drowning originate from the years 2000-2007 (Figure 1A).

Concerning the total number of citations (Figure 1B), the articles published in the year 1994 rank first with an amount of 1372 citations, followed by 1992 (1210) and 1997 (1198).

Regarding the citation rate (Figure 1C) the highest number of citations (36) is allotted to the year 1921. This sole publication concerns a new method for the diagnostic of death by drowning [19] and therefore a early new consolidated finding resulting in enormous scientific resonance [20]. Nine drowning related articles from 1970 were cited 153 times, the resultant drowning rate averages 17, followed by 16.35 and 16.33 for the years 1993 and 1994 respectively. There is an obvious decrease of the citation rates from 1997 to 2007 due to the temporal proximity to this analysis. So this must not be regarded as a loss of scientific interest in late publications on the subject drowning.

### Publication language and citation rates

**Figure 2:**
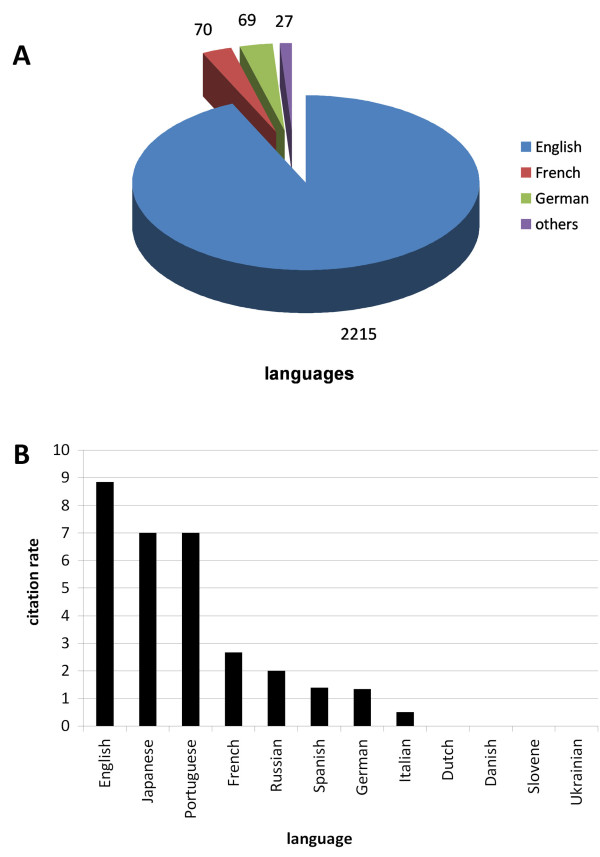
**Language analysis.** (A) total number of drowning related articles differentiated by language, (B) citation rate of publication languages.

Analysing publication languages show a notable majority (2212 of 2381 publications) of articles written in English, i.e. 93 per cent, followed by French with 70 and German with 69 though these states have a production of 106 and 145, respectively (Figure 2A). Other languages include Spanish (8), Dutch, Italian and Russian (each 4), Japanese and Portuguese (each 2), Danish, Slovene and Ukrainian (each 1). Regarding the citation rates of the named languages, English also ranks first with 8.84 citations per article, i.e. 19597 total citations (Figure 2B), followed by Japanese and Portuguese, but a closer look - which is necessary to understand the context - reveals only two publications for each language. So these citation rates are not as significant as the citation rates for English, French or German language. The drowning related articles written in Dutch, Slovene and Ukrainian have not been cited, according to this, the citation rate is 0.0.

### Country research analysis and WHO data

**Figure 3:**
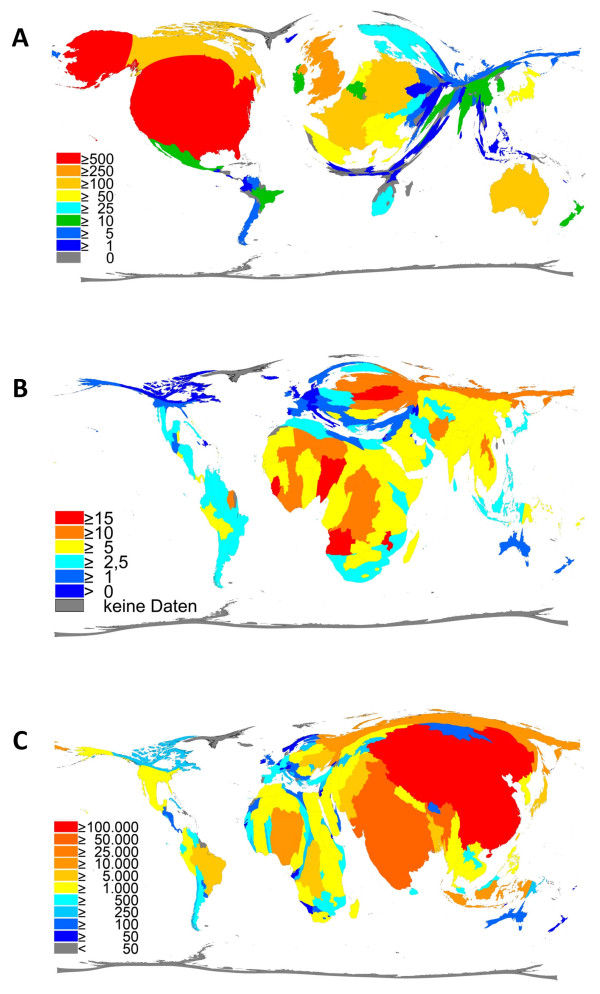
**Density equalizing calculations**. (A) Map illustrating the total number of drowning related publications for each country for the period 1900-2006. (B) Map visualizing the worldwide drowning rates in 2002. (C) Map showing the total number of drowning deaths worldwide in 2002.

The United States of America is the country with the highest output of articles on drowning, with a total of 800 publications, i.e. 34.2 per cent. The United Kingdom is in second position with 233 articles, followed by Germany with 145 publications. Density equalizing mapping was utilized to exemplify the research output by territorial resizing (Figure 3A). In this visualization, the USA evidently dominates the cartogram whereas large parts of Africa, Eastern Europe and Asia are minimized. Regarding the worldwide drowning rates (Figure 3B) there are eight African countries among the top ten countries with the highest drowning rate, Angola ranking first with 17.9 drowning deaths on 100,000 inhabitants. The average European drowning rate is 3.4, mainly contributed by Eastern European states such as Latvia (13.7), Lithuania (13.2) and Estonia (10.5). The worldwide drowning rate adds up to 5.3. But also minimized states exhibit a high number of drowning victims, e.g. Japan's drowning rate is "only" 4.6 which represents (at a population of 130 millions) 6000 (!) drowning victims for only the year 2002. To provide a better understanding of the worldwide situation of drowning, also the total number of drowning deaths is visualized (Figure 3C). Particularly China (113,000) and India (69,000) emerge from the cartogram. Also Africa is broadened due to many states with more than 1000 drowning victims. In comparison, Central America, Canada (295), Australia (238) and Western Europe are clearly minimized.

### Country research network analysis

**Figure 4:**
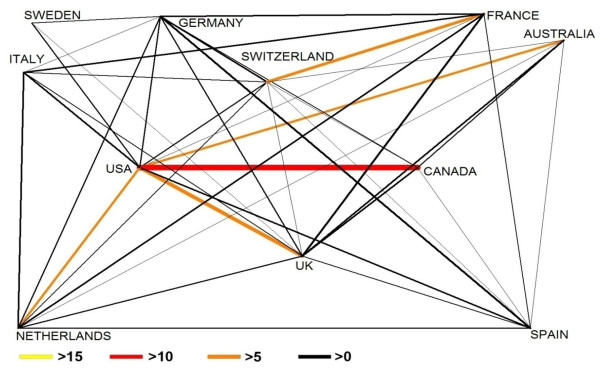
**Country network analysis**. Chart technique visualizing bilateral networking between countris for the overall number of collaborations between the two countries. Size and color of bars encode the number of bilateral cooperation.

The analysis of the international research cooperatin revealed a considerable network of global collaborations (Figure 4). A dense network of internatinal research cooperation was identified, mainly formed by North American and European countries. It was found that with a number of 15 bilateral cooperation articles, Canada and the United States are the leading cooperating countries, closely followed by the cooperation between the United States and the United kingdom (10). The USA published six articles on drowning each with Australia and the Netherlands. Regarding the total number of cooperation articles, the USA ranks first with a number of 73 cooperations, followed by the United Kingdom (49), Germany (34) France (30), Canada (28), the Netherlands (20), Switzerland (18), Australia (16), Spain (16), Italy (15) and Sweden (11). The states that produced less than ten publications in at least bilateral cooperation are not shown. To visualize research networking for drowning related articles, the chart technique was employed.

### Institutional productivity and corresponding drowning rate

**Figure 5:**
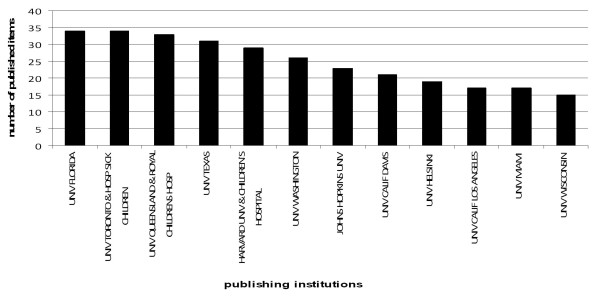
**Institution publication analysis**. Ten most productive institutions with corresponding number of published drowning related articles.

With a total number of 34 relevant publications, the University of Florida and the Canadian University of Toronto & Hospital for Sick Children rank first, followed by the Australian University of Queensland and the associated Royal Children's Hospital (33 articles). With 31 drowning related articles, another US-American institiution, the University of Texas, comes in third. Since nine of the twelve most productive institutions are in the US, this international ranking is quasi a US-national one, a fact that is consonant with the USA being the most productive country. The University of Helsinki is the only European institution in this ranking list (Figure 5).

The *Centers for Disease and Control and Prevention (CDC) *is a public authority of the United States of America which has available drowning rates of single federal states from 1989 to 1998 [21]. Comparing the US-national institutions' locations and the corresponding drowning rates of the respective states reveals that the majority of drowning rates of the most productive states (Florida, Texas, Washington) exceed the average nationwide drowning rate. Presumably due to the higher incidence of drowning accidents in turn probably due to bordering with the open sea more research effort is made on this public health care problem.

This correlation of water exposure and higher drowning rates can also be transferred to Finland, the "land of the thousand lakes" which has the highest drowning rate of all member states of the European Union in 2002 (2.5) and the highest research productivity (19) on drowning in Europe.

### Journal analysis and impact factor

**Figure 6:**
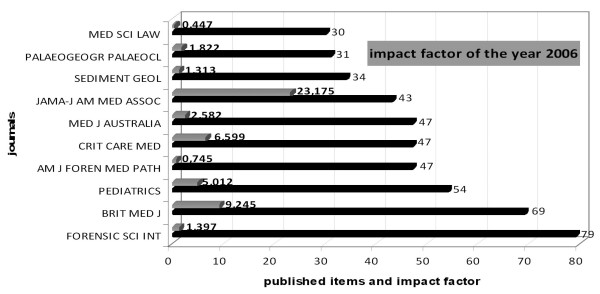
**Journal analysis**. Ranking of the most productive journals and impact factor of the year 2006.

The highest number of drowning related articles were published in the journal *Forensic Science International *(79), a journal that publishes original contributions in the many different scientific disciplines pertaining to the forensic sciences. This journal's impact factor was 1.397 in the year 2006 (Figure 6). Second ranks the *British Medical Journal *with 69 publications (impact factor 9.245), followed by *Pediatrics *(54). The highest impact factor (23.175) of the ten most publishing journals on the subject drowning holds the *JAMA (Journal of the American Medical Association).*

### Author analysis

**Figure 7:**
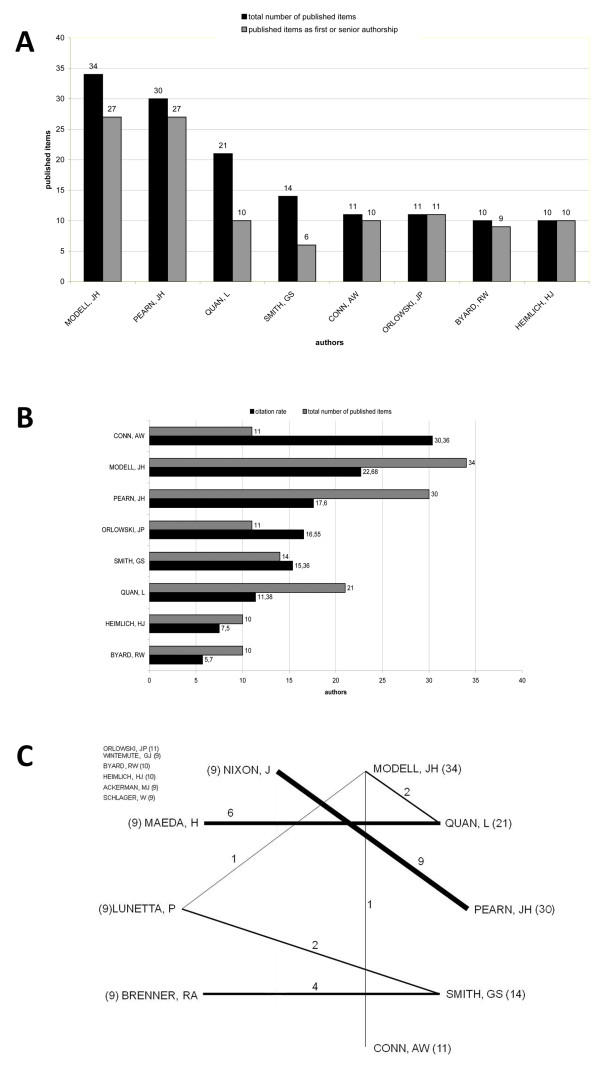
**Author analysis.** (A) Ranking of the most productive authors and first and seniorauthorship, (B) Number of publications and citation rate, (C) Chart technique visualizing the overall number of cooperation articles between at least two authors. The number of cooperation articles is written on the bars, the total number of published articles is written next to the name in brackets.

In a last step, an author analysis was performed for the 2381 drowning related articles. An author's scientific performance on the subject of drowning can be evaluated with the aid of bibliometric parameters such as the number of publications, amount of being first or senior author and citation rates. Jerome H. Modell who has been researching on drowning for several decades now was identified to be the author with the highest number of articles related to drowning (34), displaying also the highest number of first and senior authorships (27) together with Pearn who is also second in total productivity with 30 published articles (Figure 7A). James P. Orlowski and Henry J. Heimlich hold a first or senior authorship in all their articles on drowning. Regarding the origin, five authors are from the USA (Modell, JH, Quan, L, Smith, GS, Orlowski, JP and Heimlich, HJ). Pearn, JH, as well as Byard, RW operate in Australia. Only Conn, AW is Canadian. Concerning the citation rate, Conn, AW ranks first with a citation rate of 30.36 (Figure 7B). Analysing his work shows that four relevant articles hold more than 50 citations each. The main contribution to Jerome H. Modell's citation rate is an clinical review [9] with a total of 108 citations. Regarding Pearn, JH (17.6), Orlowski, JP (16.55), Smith, GS (15.36) and Quan, L (11.38), four scientists hold a citation rate between ten and 20. The average citation rate of all drowning related publications listed in the ISI-Web of Science database from the period 1900-2006 is 7.96.

Visualizing the cooperations between authors, the chart technique is used (Figure 7C). A minimum of nine cooperation articles is determined. Nixon, J and Pearn, JH produced nine drowning related articles together. They are all published by the Australian Childrens Hospital and the University of Queensland and date from the period 1976-1986. The six cooperation articles from Maeda, H and Quan, L all originate from Japan and are all related to the category Medicine, Legal which represents a thematic focus of the drowning related publications from this nation.

## Discussion

After road traffic injuries, drowning is the second leading cause of unintentional injury death worldwide [22] and therefore a constant problem of the global burden of disease [1]. The past decades of research in the field of drowning have been challenged by revolutionary insights in pathogenesis and therapy of drowning victims. Despite its continuing actuality and the notable effort in drowning research, it is still a doggerel medical topic with only few preventive medical approaches. Therefore, further investigations are necessary in order to enable and promote understandings concerning the condition drowning. The present study represents the first bibliometric review of worldwide research activities on the subject of drowning.

Regarding the results of the language analysis, it is confirmed that with English as international voice of medicine it is possible for scientists to attract a big audience whereas other languages experience a loss of importance [23,24]. The subsequent rise of impact factors of English language journals results in turn in more citations which leads to a language bias [25] which is also supported by the fact that the ten most productive journals publishing on the subject drowning are in English language without exception. Analyzing the cartograms, productive countries, e.g. the USA and European states, show low drowning rates compared to developing and industrializing states, e. g. Asian and African countries, where most drowning incidents occur. It could be concluded that the amount of drowning rates and the the total number of drowning victims reflect a global marker of deficits in health and social policies. In first world countries there are a better public education and perception on drowning risks, more efficient preventive measurements and better therapeutic options. This could be a one possible explanation for the low number of drowing victims in first world countries. The author analysis showed that an author's research producitvity (number of publications) does not necessarily correlate with the scientific calue of the author's work (citation rate). Consonant with the leading role of the USA in reseraching drowning in general, more than half of the most productive authors originate from this nation.

It has to be considered that the analysis of drowning related artivles in the present study cannot be regarded as completely representative of global occupational research activity in this filed, since the data was retrieved from only one database (Web of Science), denoting a potential bias. Whereas the Web of Science is among the largest global biomedical databases, there are still publications which cannot be traced by the use of this system. Nonetheless, it can be hypothesized that the present findings represent common trends in the research of drowing. In addition, the employed quality indicators need to be regarded critically and therefore, the data should not be over interpreted as indicated by numerous previous articles [26-28]. Furthermore, the drowning data retrieved from the WHO do not exactly display the actual number of drowning victims. According to estimations, it is much higher because the number of near-drowning victims [1] and drownings due to cataclysms, water and other transport accidents, assaults and suicide are excluded [22]. Also the clinical symptoms cannot be related to a certain disease according to the ICD because of the huge variability resulting in a complicated documentation because the chain of causation is unclear: For example, death from drowning would also be the ruling for a drowing victim who develops and dies from aspiration pneumonia after being stable withsevere hypoxic encephalopathy for weeks to months. However, if that same patient died of acute myocardial infarcion, it most likely would be classified as a death not related to drowning [29]. Another reason for underreporting is a lack of standardized definitions within the terminology of drowning [30]. The new, more appropriate, world-wide uniform definition of drowning demanded was finally presented on the World Congress on Drowning in 2002: "Drowning is the process of experiencing respiratory impairment from submersion/immersion in liquid" [31]. This definition was also seized for the "Utstein Style", i. e. recommended guidelines for uniform reporting of data from drowning [29] to provide adaquate and reliable international registrations of drowning incidents.

It is interesting to compare the present results to other studies in the field of health geographics recently published [32,33]. For instance, Boulos published a study on geography and medical journalology. He focussed on the geographical distribution of articles published in the leading medical informatics journal Medical Informatics & the Internet in Medicine between 1999 and 2004 [32]. It was shown that the examined journal had an international outreach, with authors from 24 countries, spanning four continents [32]. European articles with 81.25% of all articles counted dominated the study. In specific, articles from the UK (15.63%) and Greece (15.63%) were numerous [32]. There were no contributions from Africa or South America present. This finding within the field of medical informatics is differing to the present results found for the field of drowning research in which the United States of America is the country with the highest output of articles.

A further study published by Uthman and Uthman focussed on the research activity in a singly continent, Africa [33]. Analysing articles indexed by PubMed between 1996 and 2005 it was demonstrated that biomedical research production in Africa is highly skewed [33]. South Africa, Egypt, and Nigeria were found to make up a striking 60% of the total number of articles. The authors also adjusted for population size and reported that smaller countries, such as The Gambia, Gabon and Botswana, were more productive in relation to Nigeria and Kenya [33]. The Gambia and Eritrea had better records when total production was adjusted for gross domestic product. The contribution of Africa to global research production was persistently low through the period studied [33]. Our present data in the field of drowning research is in aggreement with this specific set of data recorded only for Africa and biomedical research activity.

## Conclusion

The present study represents a first detailed scientometric Web of Science database analysis and visualization of drowning related publications using density equalizing calculations and chart techniques. The proposed aims of the studies were to analyse total numbers of published items and citations, country total number of published items in comparison to worldwide total number and rate of drowning deaths, country research network parameters, institutional productivity and author analysis. It can be concluded that interest and feedback on the part of the research community on the subject drowning have been rising continuously in the past three decades. Assumingly, according to the actuality of drowning incidents, e.g. due to natural catastrophes, and the climate change, he scientific turning to the topic drowning will achieve even greater dimensions. The techniques established here can be of use for future bibliometric studies in this field. Ideally, these techniques should be combined with recently published plattforms such as HEALTH GeoJunction. This is a geovisual analytics-enabled web application providing computational reasoning methods to extract place-time-concept information from bibliographic data [34].

## Competing interests

The authors declare that they have no competing interests.

## Authors' contributions

DG and BK have initiated the study, supervised the project and participated in the analyses and interpretation. US has carried out the main data analysis and interpretation within her doctoral thesis. CS has written the analysis program. SU, SZ, DM and DK have participated in the discussion of the results and drafting of the manuscript. All authors have read and approved the final manuscript.
